# Biomimetic Black Phosphorus Nanosheet-Based Drug Delivery System for Targeted Photothermal-Chemo Cancer Therapy

**DOI:** 10.3389/fbioe.2021.707208

**Published:** 2021-08-19

**Authors:** Jinxiu Cao, Junyang Qi, Xun Lin, Yue Xiong, Fumei He, Wenbin Deng, Gan Liu

**Affiliations:** School of Pharmaceutical Sciences (Shenzhen), Sun Yat-sen University, Guangzhou, China

**Keywords:** biomimetic nanoparticles, black phosphorus, drug-self-stabilization, mesenchymal stem cell membrane, combined chemo-photothermal cancer therapy, targeted delivery

## Abstract

As a biodegradable material, black phosphorus (BP) has been considered as an efficient agent for cancer photothermal therapy. However, its systemic delivery faces several hurdles, including rapid degradation in blood circulation, quick clearance by the immune system, and low delivery sufficiency to the tumor site. Here, we developed a biomimetic nanoparticle platform for *in vivo* tumor-targeted delivery of BP nanosheets (BP NSs). Through a biomimetic strategy, BP NSs were utilized to coordinate with the active species of oxaliplatin (1,2-diaminocyclohexane) platinum (II) (DACHPt) complexions, and the nanoparticles were further camouflaged with mesenchymal stem cell (MSC)–derived membranes. We showed that the incorporation of DACHPt not only decelerated the BP degradation but also enhanced the antitumor effect by combining the photothermal effect with chemotoxicity. Furthermore, MSC membrane coating increased the stability, dispersibility, and tumor-targeting properties of BP/DACHPt, significantly improving the antitumor efficacy. In short, our work not only provided a new strategy for *in vivo* tumor-targeted delivery of BP NSs but also obtained an enhanced antitumor effect by combining photothermal therapy with chemotherapy.

## Introduction

Owing to its distinctive structure and excellent physicochemical properties, black phosphorus (BP), an emerging member of the two-dimensional nanomaterials family has attracted extensive research interests in cancer photothermal therapy (Luo G. et al., 2019; Gao, et al., 2020; Hu, et al., 2020; Qi, et al., 2020). Compared with existing nanomaterials such as graphene and MoS_2_, BP offers a much larger surface-to-volume ratio due to its puckered lattice configuration (Ray 2016) and, therefore, has great potential as a superior drug nanocarrier, especially for cancer combination therapies (Luo M. et al., 2019; Wang, et al., 2020). Moreover, BP can easily degrade into nontoxic phosphorus compounds, such as phosphate, phosphonate, and other P_X_O_Y_ (Huang, et al., 2016; Zhang, et al., 2018), showing good biodegradability and safety *in vivo* (Shao, et al., 2018; Xue, et al., 2020). However, *in vivo* adoption of BP faces several hurdles, such as rapid degradation in blood circulation (Abate, et al., 2018), quick clearance by the immune system (Qu, et al., 2017), and low delivery sufficiency to the tumor site (Mitchell, et al., 2021).

Although multiple modification strategies have been developed for BP nanosheets (BP NSs) to improve their therapeutic effect, developing a stable and targeted BP NS-based multifunctional drug delivery system is still a challenging task. For example, as a common method to modify BP NSs, the PEGylation can effectively reduce their aggregation and keep them stable (Ou, et al., 2018; Wan, et al., 2020), but this electrostatic adsorption of polyelectrolytes onto BP NSs is instable upon intravenous administration due to the desorption. On the other hand, although chemical modification is a valid way to enhance the ambient stability of BP NSs by passivating their surface P atoms, it cannot improve the dispersion of BP NSs, leading to quick clearance of BP by the immune system. For example, we previously used the active species of oxaliplatin (1,2-diaminocyclohexane) platinum (II) (DACHPt) complexion to coordinate with exposed lone pair electrons of BP NSs (Liu G. et al.,. 2019). This strategy markedly stabilized BP NSs and meanwhile introduced chemotherapeutics DACHPt with a high loading efficiency, which showed more significant synergistic antitumor effects. However, in a complex physiological environment, this is still not an ideal delivery system requiring BP protection from immune attack and targeted delivery at tumor sites.

Recently, owing to its ability of directly inheriting complex membrane surface molecules from the source cell, the cell membrane camouflaging of nanoparticles has emerged as an attractive strategy for imparting nanoparticles a wide range of functionalities, including a prolonged circulation time (Wang, et al., 2018), modulated immune responses (Gao, et al., 2015), and specific targeting (Zhang, et al., 2018). Among the popular cell candidates, mesenchymal stem cells (MSCs) have received close attention due to their unique migration ability and intrinsic tumor tropism, which are primarily mediated by diverse surface receptors on their membranes (Momin, et al., 2010). In addition, ease of isolation from *in vitro* culture (Kusuma, et al., 2015) and safety in allogeneic transplantation (Noriega, et al., 2017; Tsumanuma, et al., 2016) are also the advantages of MSCs to be the source of membrane. Based on these properties, MSC membrane-coated nanoparticles have obtained a very good effect in targeting tumor and prolonging circulation time (Gao, et al., 2016a; Gao, et al., 2016b).

**FIGURE 1:**
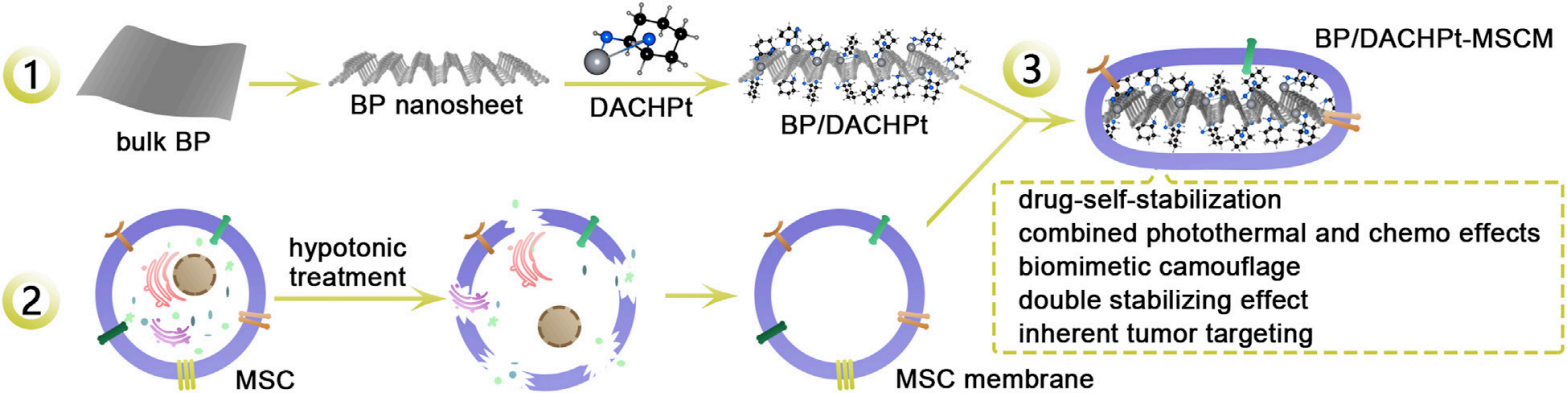
Schematic showing the preparation of biomimetic BP/DACHPt-MSCM nanoparticles for targeted photothermal-chemo cancer therapy. 1: BP NSs prepared with bulk BP coordination with DACHPt to synthesize BP/DACHPt nanoparticles. 2: Extraction of MSC membranes by hypotonic treatment. 3: Ultrasonication mediated self-assembly of MSC membrane on the surface of BP/DACHPt nanoparticle to yield BP/DACHPt-MSCM nanoparticles.

Accordingly, we designed a biomimetic tumor-targeted BP nanoplatform, called as BP/DACHPt-MSCM (Figure 1). In this nanoplatform, the active species of platinum-based anticancer drugs (DACHPt) was utilized to coordinate with BP NSs to form BP/DACHPt nanoparticles, and it was further camouflaged with MSC-derived membrane. We demonstrated that BP/DACHPt-MSCM had good dispersibility, much enhanced stability, and efficient tumor targeting. *In vitro* cytotoxicity results showed the combination of chemotherapy and photothermal therapies resulted in an excellent ability to inhibit the tumor cell growth.

## Materials and Methods

### Materials

The bulk BP was purchased from Nanjing MKNANO Technology Co., Ltd. (Nanjing, China). DACHPtCl_2_, N-methyl-2-pyrrolidone (NMP), and fluorescein isothiocyanate (FITC) were purchased from Macklin Biochemical Co., Ltd. (Shanghai, China). AgNO_3_ was purchased from J&K Chemical (Shanghai, China). Phycoerythrin (PE) anti-human CD73 antibody, PE anti-human CD90 antibody, PE anti-human CD105 antibody, FITC anti-human CD44 antibody, FITC anti-human CD45 antibody, and FITC anti-human HLA-DR antibody were purchased from Elabscience (Texas, United States). Tris-HCl was purchased from Beijing Solarbio Science & Technology Co., Ltd. (Beijing, China). Phenylmethylsulphonyl fluoride (PMSF) was purchased from Shanghai Aladdin Biochemical Technology Co., Ltd. (Shanghai, China). 1,1’-Dioctadecyl-3,3,3’,3’-tetramethylindodicarbocyanine,4-chlorobenzenesulfonate salt (DiD) was purchased from Coolaber Technology Co., Ltd. (Beijing, China). Cell Counting Kit-8 (CCK-8) was purchased from ApexBio (Houston, United States). Dead Cell Apoptosis Kit (Annexin V-FITC) and propidium iodide (PI) were purchased from Jiangsu KeyGEN Biotechnology Co., Ltd. (Jiangsu, China). Fetal bovine serum (FBS), high-glucose Dulbecco’s modified eagle medium (H-DMEM), alpha minimum essential medium (α-MEM), penicillin–streptomycin, trypsin-EDTA, and phosphate buffer saline solution (PBS) (pH 7.4) were obtained from Gibco Life Technologies (AG, Switzerland). All other commercially available chemicals and reagents were used as received.

### Synthesis and Characterization of BP NSs

To prepare BP NSs, the bulk BP was ground to powder in a mortar, which was then dispersed in NMP (2 mg/ml) and sonicated for 4 h by a probe sonicator (on/off cycle: 5 s/5 s and 25% power) in an ice bath, followed by ultrasonication with a Kunshan KQ-600 GDV bath sonicator for 10 h (frequency: 40 kHz, power: 300 W) to perform liquid exfoliation. Next, the solution was centrifuged for 15 min at 7,000 rpm and the supernatant containing BP NSs was collected. Afterward, the supernatant was centrifuged for 15 min at 12,000 rpm and the precipitate was dispersed in NMP for further use.

Transmission electron microscopy (TEM) was performed using an FEI Tecnai G2 Spirit microscope operated at 120 kV. Atomic force microscopy (AFM) was carried out using the Bruker Dimension Fastscan microscope.

### Synthesis and Characterization of BP/DACHPt

BP/DACHPt was prepared as per our method described previously (Liu M. et al., 2019). Briefly, AgNO_3_ (10 mM) was mixed with DACHPtCl_2_ in DI water (molar ratio: AgNO_3_/DACHPtCl_2_ = 2) in the dark at room temperature (RT) by stirring for 24 h. After the reaction, the mixture was centrifuged to remove generated AgCl precipitate. The supernatant was filtered through a micropore film and freeze-dried. Next, BP NSs dispersed in NMP (2 mg/ml) was mixed with DACHPt (mass ratio: DACHPt/BP NSs = 2) in the dark at RT for 12 h. Then, BP/DACHPt was harvested by centrifugation and washed with deionized (DI) water.

Raman spectroscopy was conducted using a Renishaw inVia Qontor confocal Raman microscope with the wavelength laser of 532 nm as the light source. X-ray photoelectron spectroscopy (XPS) was performed using the ESCALab250 spectrometer with Al Kα radiation.

### Cell Culture

MSCs, derived from human placental chorionic villi, were cultured in α-MEM supplemented with 10% FBS. The A549 cells were incubated in H-DMEM containing 1% penicillin–streptomycin and 10% FBS. All cells were maintained at 37 C in a humid atmosphere with 5% CO2.

### Characterization of MSCs

The bright-field image of MSCs was obtained by a NIKON ECLIPSE Ti2 fluorescence microscope. The immunophenotype of MSCs was estimated by Beckman Coulter CytoFLEX flow cytometry (FACS). Briefly, the cells were collected and stained for 30 min at 4 C with FITC-labeled or PE-labeled monoclonal antibodies against CD73, CD90, CD44, CD105, CD45, and HLA-DR.

### Derivation and Characterization of MSC Membranes

To generate MSC membranes, MSCs were harvested by scraping and cleaned twice with PBS by mild centrifugation at 700 g for 5 min. The cells were resuspended in a hypotonic buffer containing 10 mM Tris-HCl and 1 mM PMSF. The cell suspension was homogenized in an ice bath with a Dounce homogenizer for 10 min. Next, the solution was centrifuged at 4,000 g for 10 min, and the collected supernatant was recentrifuged at 20,000 g for 30 min. The obtained MSC membranes were once washed with PBS and suspended in PBS with PMSF for further use. The TEM image was performed using a Hitachi HC-1 microscope operated at 80 kV.

### Preparation and Characterization of MSC Membrane-Coated BP/DACHPt Nanoparticles (BP/DACHPt-MSCM)

To synthesize biomimetic BP/DACHPt-MSCM nanoparticles, BP/DACHPt dispersed in PBS was mixed with MSC membranes in a mass ratio of 4:1. Notably, the concentration of MSC membranes was represented by that of the membrane protein measured with the BCA assay. After vortexing for 1 min, the mixture was sonicated for 5 min by a bath sonicator (frequency: 40 kHz, power: 100 W) to yield BP/DACHPt-MSCM nanoparticles.

TEM images were performed using an FEI Tecnai G2 Spirit microscope operated at 120 kV. The zeta potentials were determined using the NanoBrook 90PlusPALS phase analysis light scattering. Confocal microscopy images were obtained by a NIKON ECLIPSE Ti2 fluorescence microscope.

### Stability Study of BP Formulations

To test the changes in the hydration particle size and UV–vis absorption during the degradation process of three BP formulations (BP NSs, BP/DACHPt, and BP/DACHPt-MSCM), these BP formulations with the same internal BP concentration were pre-dispersed in air-exposed water for different periods. At each time point, the hydration particle size of the samples was measured by using the NanoBrook 90PlusPALS phase analysis light scattering and the UV–vis absorption from 400 to 850 nm of the samples was measured by using the PerkinElmer LAMBDA 365 UV/Vis spectrophotometer. To acquire the TEM images of BP/DACHPt-MSCM after NIR irradiation, the BP/DACHPt-MSCM dispersed in DI water was irradiated with 808 nm NIR laser (power density: 1.0 W/cm^2^) for 10 min followed by dripping onto the surface of a copper grid and the TEM images were obtained using an FEI Tecnai G2 Spirit microscope operated at 120 kV. To test the photothermal stability of BP NSs, BP/DACHPt, and BP/DACHPt-MSCM, these three BP formulations having 30 μg/ml of BP were pre-dispersed in air-exposed water by stirring for different time durations (0, 12, 24, 48, and 72 h). At each time point, the photothermal conversion ability of the distinct BP formulations was tested by monitoring their temperature changes using a Fluke Ti450 infrared thermal imaging camera under Shanxi KaiSite KS-810F-8000 808-nm NIR laser irradiation (power: 1.0 W/cm^2^, duration: 10 min). The dispersity of BP NSs, BP/DACHPt, and BP/DACHPt-MSCM nanoparticles that had the same amount of BP was evaluated in DI water, PBS, and DMEM containing 10% FBS for 12 h at RT.

### Cellular Uptake of Nanoparticles

The cellular uptake of nanoparticles was estimated by FACS using CytExpert software. Briefly, the bare or MSC membranes coated FITC-conjugated BP/DACHPt nanoparticles were incubated with the A549 cells for 1, 2, 3, or 4 h, respectively. In addition, for green fluorescent dye FITC loading, 1 mg/ml of BP/DACHPt was mixed with FITC (10 μg/ml) in NMP and then stirred at room temperature for 12 h. The excess unloading was washed away via rinsing with NMP and DI water. Then, the cells were washed thrice before collection and the fluorescence was estimated by FACS.

### CCK-8 Assay

The Cell Counting Kit-8 (CCK-8) assay was used to quantify the antitumor effect of freshly prepared BP-based formulations by estimating the viability of A549 cells. The A549 cells (1 × 10^4^ cells/well) were seeded into 96-well plates and cultured overnight. Then, the cells were cultured with BP, BP/DACHPt, or BP/DACHPt-MSCM (having internal BP concentration of 0, 1.875, 3.750, 7.500, 15.000, and 30.000 μg/ml) for 24 h without any irradiation. On the other hand, the A549 cells in the NIR irradiation group were incubated with the concentration of 0, 1.875, 3.750, 7.500, 15.000, and 30.000 μg/ml for 4 h followed by irradiating with 808 nm NIR laser (power density: 1.0 W/cm^2^) for 10 min. Then, the cells were cultured for another 8 h at 37 C. Last, the CCK-8 solution was added to each well and the absorbance was measured at 450 nm by a PerkinElmer VICTOR NivoTM Multimode Plate Reader.

To assess the effect of combined therapy of DACHPt and BP, we calculated the combination index (CI) (Chou 2010) with respect to experimental parameters (IC_50_) by using the formula CI = C_DACHPt,50_/IC_50,DACHPt_ + C_BP,50_/IC_50,BP_. C_DACHPt,50_ and C_BP,50_ refer to the drug concentration of DACHPt and BP when 50% cell inhibition was induced in the combined group. IC_50, DACHPt_ and IC_50, BP_ are the drug concentrations resulting in 50% inhibition with a single DACHPt or BP. The concentration of DACHPt and BP in BP/DACHPt was calculated after quantifying their concentration of Pt and P elements by Thermoscientific inductively coupled plasma mass spectrometry. CI allows quantitative determination of drug interactions, where CI < 1, = 1, and >1 indicate synergism, additive effect, and antagonism, respectively.

### Calcein-AM/PI Assay

The antitumor effect of freshly prepared BP formulations at a certain concentration was tested by the Calcein-AM/PI assay. Briefly, the A549 cells (1 × 10^4^ cells/well) were seeded into 96-well plates and cultured overnight. Subsequently, the cells were incubated with BP, BP/DACHPt, or BP/DACHPt-MSCM (an internal BP concentration of 7.5 μg/ml) for 24 h without irradiation. On the other hand, the A549 cells in NIR irradiation group were incubated with the internal BP concentration of 15.0 μg/ml for 4 h followed by 808 nm NIR irradiation (power density: 1.0 W/cm^2^, duration: 10 min) and further incubated for another 8 h at 37 C. Afterward, to explore the antitumor effect, the cells were co-stained with Calcein-AM/PI by a NIKON ECLIPSE Ti2 fluorescence microscope.

## Results and Discussion

### Synthesis and Characterization of BP NSs and BP/DACHPt

**FIGURE 2:**
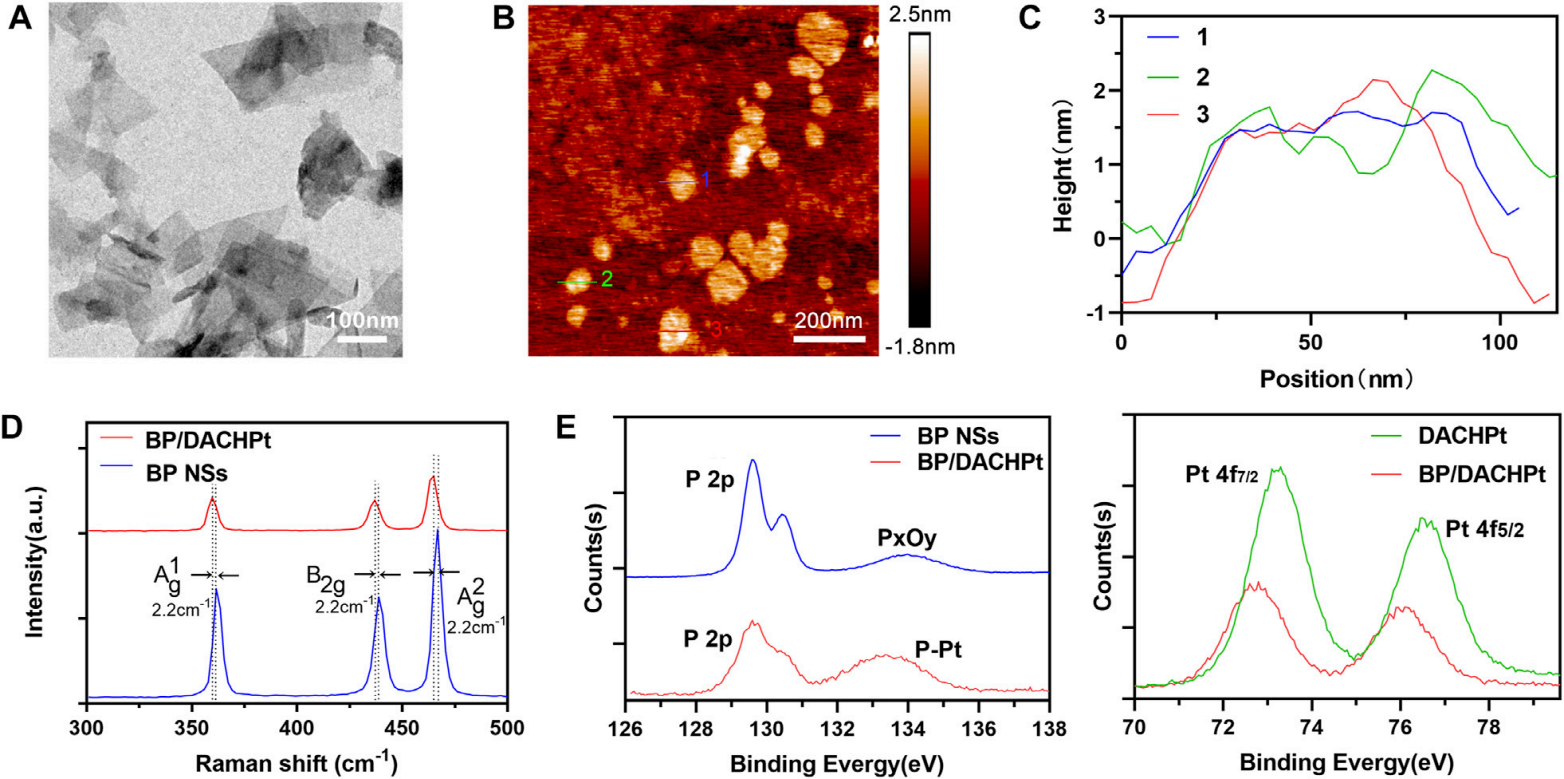
Characterization of BP NSs and BP/DACHPt. **(A)** Transmission electron microscopy (TEM) image of BP NSs. **(B)** Atomic force microscopy (AFM) image of BP NSs. **(C)** Height profiles of the three randomly chosen lines in B. **(D)** Raman spectra of BP/DACHPt and BP NSs. **(E)** HR-XPS spectra of P 2p and Pt 4f.

The morphology of BP NSs prepared by a liquid exfoliation method was observed by TEM and AFM. As shown in the TEM image (Figure 2A), BP NSs exhibited a nanosheet structure with the size of 100–400 nm. The topographic morphology of BP NSs was shown in AFM image (Figure 2B). The heights of three randomly selected BP NSs were 0.8–2.3 nm (Figure 2C), indicating that BP NSs had few layers. Next, to confirm the interaction between DACHPt and BP NSs, the structural changes in BP/DACHPt and BP NSs were tested by Raman spectroscopy. There were three characteristic peaks in the spectrum of BP/DACHPt, relating to the one out-of-plane mode A_g_^1^ at 359.7 cm^−1^ and two in-plane modes of B_2g_ at 436.9 cm^−1^ and A_g_^2^ at 465.0 cm^−1^ (Figure 2D). In contrast to BP NSs, the A_g_^1^, B_2g_, and A_g_^2^ peaks of BP/DACHPt showed a red-shift of about 2.2 cm^−1^. This indicated that the Pt–P bonds suppressed the oscillation of surface P atoms decreasing the corresponding scattering energy. The Pt–P coordination was further evaluated by X-ray photoelectron spectroscopy (XPS), while BP NSs and DACHPt were used as controls. The P 2p core level spectrum of BP NSs showed three peaks, corresponding to P 2p_3/2_ at 129.58 eV and P 2p_1/2_ at 130.43 eV which were the characteristic peak of BP crystal (Ni, et al., 2018) (Figure 2E). In addition, the oxidized P species (P_x_O_y_) sub-bands is located at 133.98 eV, indicating the slight oxidation of BP NSs surface (Tao, et al., 2017). Accordingly, the P 2p peak at 129.60 eV of BP/DACHPt is attenuated whereas the peak at 133.8 eV is enhanced, which can be attributed to the formation of P^5+^ species via the coordination between DACHPt and P atoms on the surface of BP/DACHPt. Meanwhile, the Pt 4f signals of DACHPt and BP/DACHPt were also measured. DACHPt showed two characteristic Pt peaks (4f_7/2_ and 4f_5/2_) at 73.30 and 76.50 eV (Fojtů et al., 2017). Owing to the electron donation from Pt (II) metal center to P, this set of doublets in BP/DACHPt was shifted toward lower binding energies of 72.80 and 75.95 eV. All these results verified the successful coordination between DACHPt and BP NSs.

### Characterization of MSCs and MSC Membranes

**FIGURE 3:**
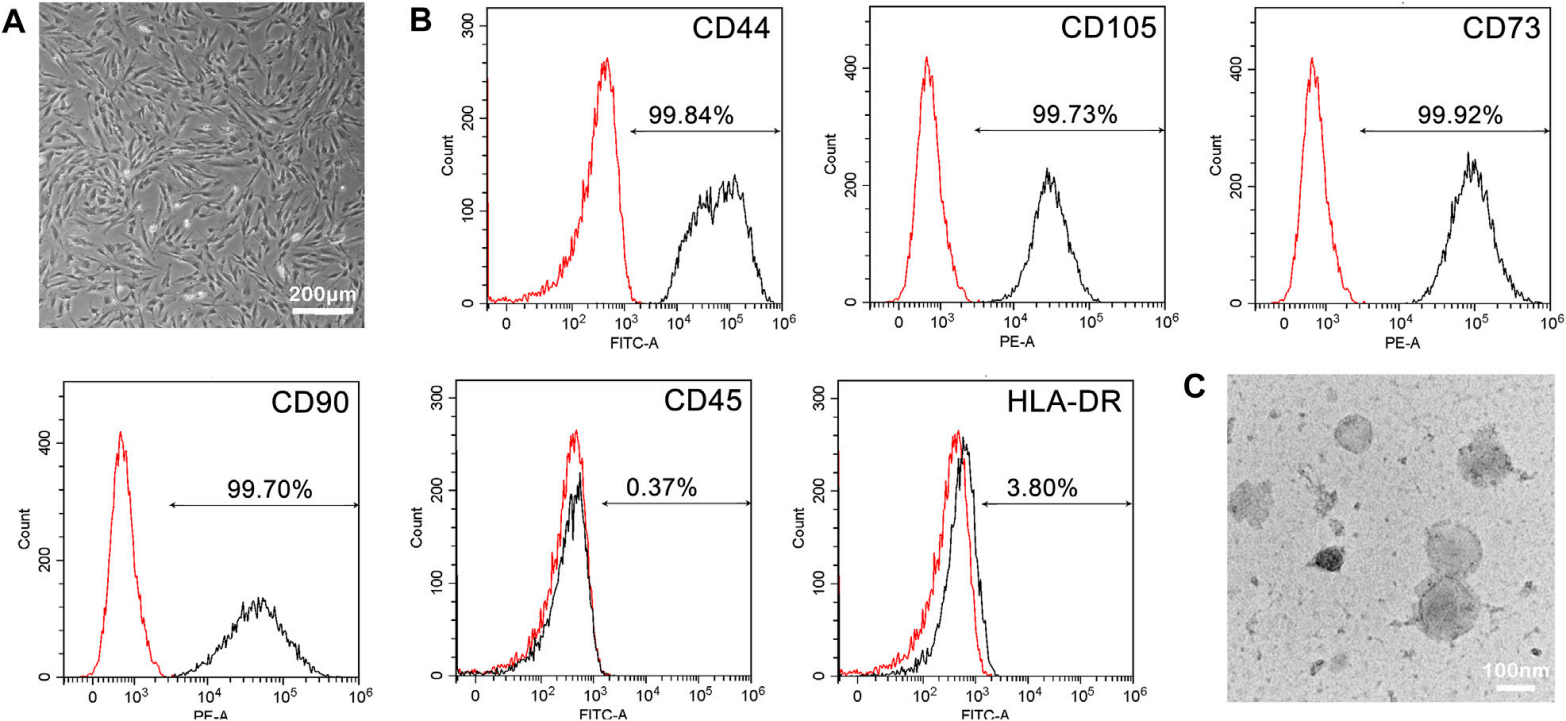
Characterization of MSCs and MSC membranes. **(A)** Bright-field microscopy image of MSCs. **(B)** Expression of MSCs cell surface markers. Histograms showing the expression of CD44, CD105, CD73, CD90, CD45, and HLA-DR. The black and red represent the MSC marker antibody and the corresponding control antibody staining, respectively. **(C)** TEM image of MSC membranes.

Considering that the real MSCs is the prerequisite for the extracted membranes to carry out expected functions, we evaluated the morphology and cell surface markers of chorionic villi-derived MSCs (CV-MSCs). In Figure 3A, CV-MSCs showed the characteristic fibroblast-like morphology as of MSCs. Furthermore, FACS analysis of phenotypic markers revealed that the cultured MSCs strongly (> 99%) expressed CD44, CD105, CD73, and CD90, while lacked expression (< 5%) of CD45 and HLA-DR surface molecules (Figure 3B). These results were in agreement with the recommendations of the MSCs by the International Society for Cellular Therapy (ISCT) (Dominici, et al., 2006), validating the reliability of MSCs source. Subsequently, MSC membranes were extracted by hypotonic treatment, followed by density gradient centrifugation. As showed in Figure 3C, the membranes exhibited a vesicle structure.

### Synthesis and Characterization of BP/DACHPt-MSCM

**FIGURE 4:**
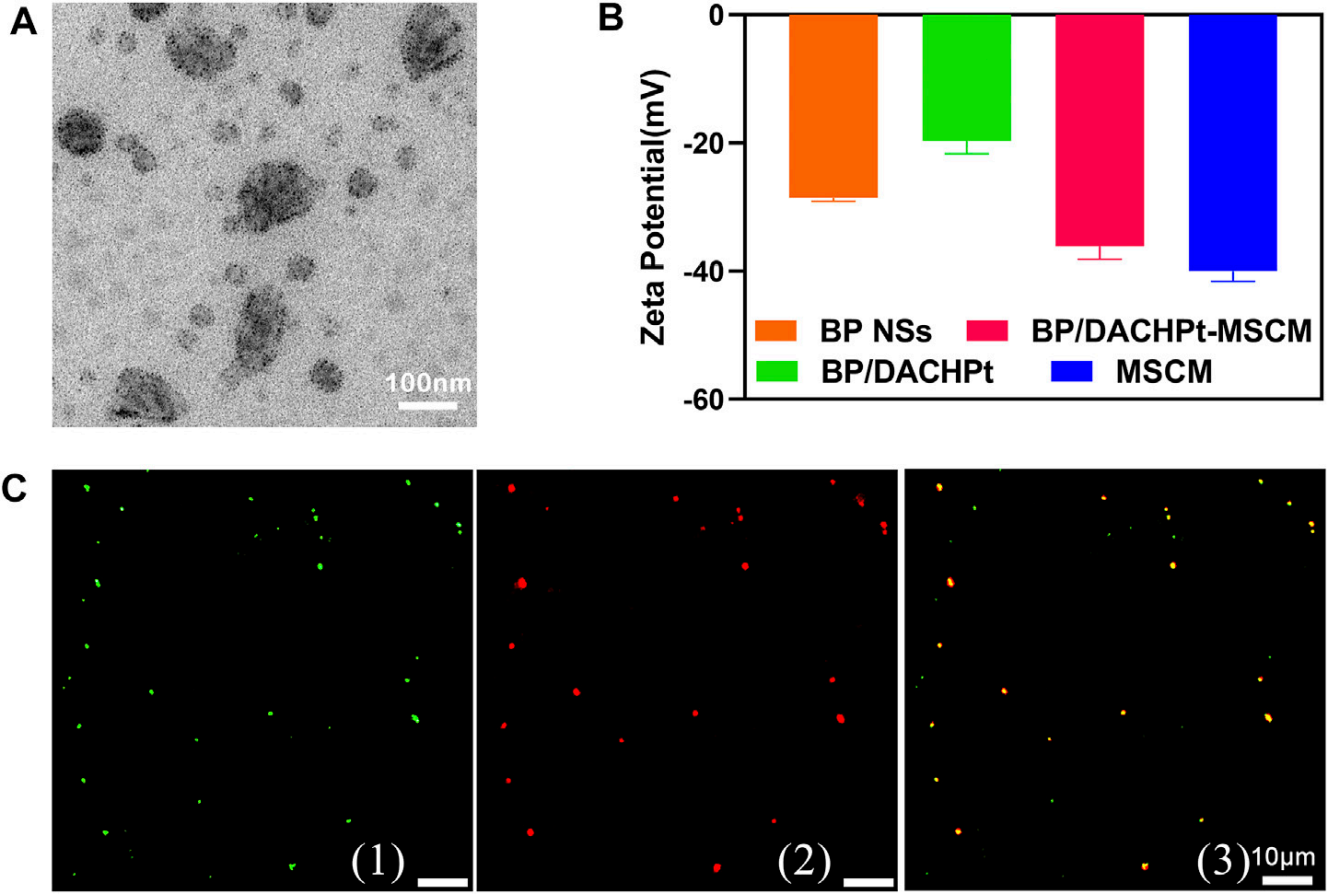
Characterization of biomimetic nanoparticles. **(A)** TEM image of BP/DACHPt-MSCM nanoparticles. **(B)** Zeta potential of BP NSs, BP/DACHPt nanoparticles, MSC membranes, and BP/DACHPt-MSCM nanoparticles. Data present mean ± s.d. (*n* = 3). **(C)** Confocal images of fluorescence-labeled BP/DACHPt-MSCM nanoparticles: (1) the green, (2) red, and (3) yellow are the fluorescence of encapsulated BP/DACHPt nanoparticles, MSC membranes, and merged fluorescence, respectively.

To prepare the MSC membrane-coated BP/DACHPt nanoparticles, MSC membranes assembly on the surface of BP/DACHPt nanoparticle was driven by ultrasonication. As showed in Figure 4A, MSC membranes completely covered the BP/DACHPt nanoparticles forming a distinctive lemma. The zeta potential characterization (Figure 4B) showed that the surface charge of BP/DACHPt nanoparticles (−19.7 mV) was lower than that of BP (−28.5 mV), while after encapsulation, the surface charge of BP/DACHPt-MSCM (−36.1 mV) increased significantly, nearly equivalent to that of MSC membranes (−40.0 mV). This clearly demonstrated the successful development of BP/DACHPt-MSCM. For visual analysis of encapsulation, BP/DACHPt nanoparticles and MSC membranes were stained with FITC and DiD, respectively, and evaluated by fluorescence microscopy. As shown in Figure 4C, the green fluorescence of BP/DACHPt was almost completely overlapping with the red fluorescence of MSC membranes, indicating a high encapsulation rate of MSC membranes.

### Stability Assessments of BP/DACHPt-MSCM Nanoparticles

**FIGURE 5:**
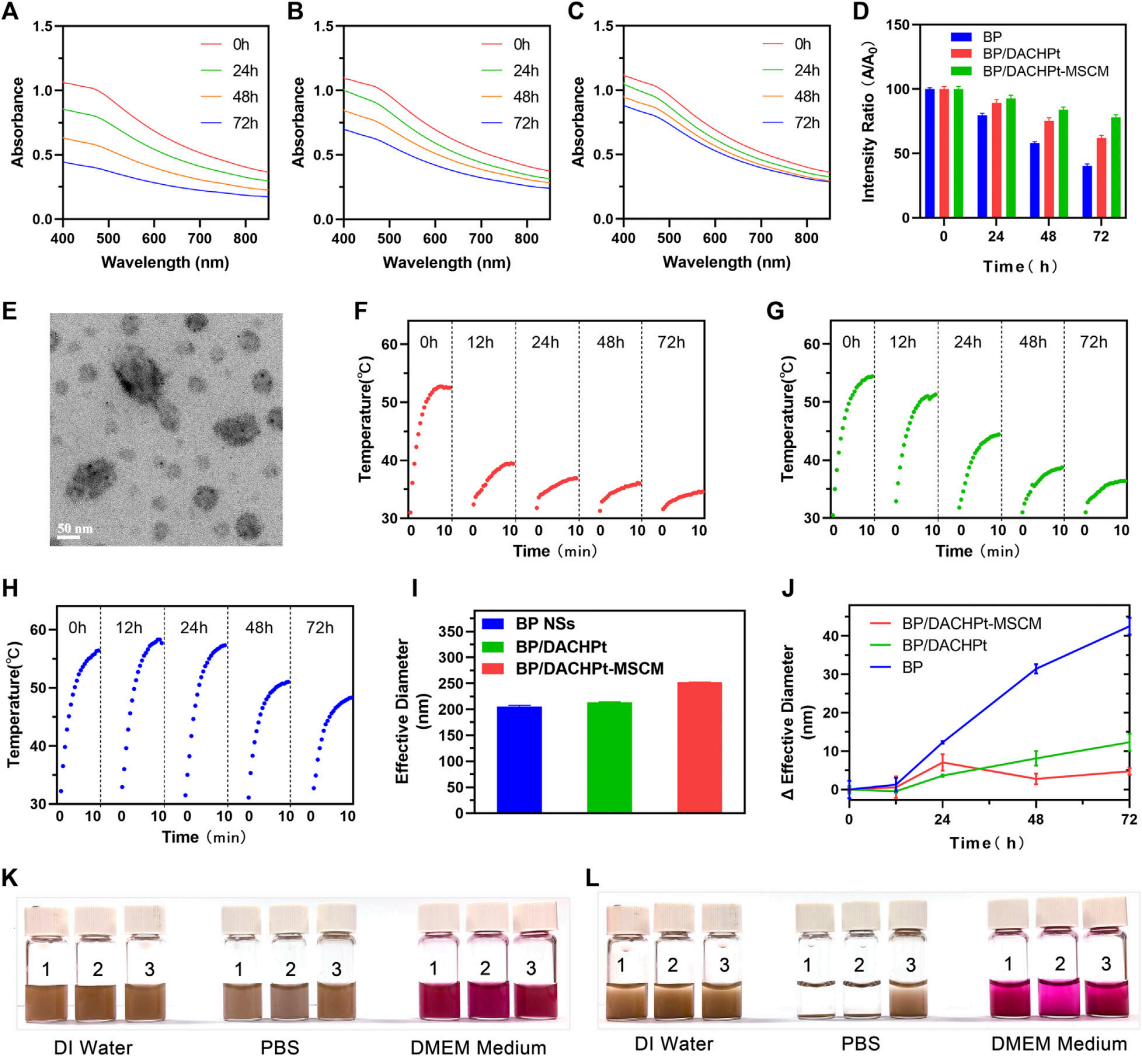
Enhanced stability of BP/DACHPt-MSCM nanoparticles. Absorption spectra of **(A)** BP NSs, **(B)** BP/DACHPt, and **(C)** BP/DACHPt-MSCM dispersed in air-exposed water for 0, 24, 48, and 72 h. **(D)** Average variation of the absorption ratios at 420, 440, and 460 nm (A/A_0_) of BP, BP/DACHPt, and BP/DACHPt-MSCM. **(E)** TEM image of BP/DACHPt-MSCM with NIR irradiation. Photothermal heating curves of **(F)** BP NSs, **(G)** BP/DACHPt, and **(H)** BP/DACHPt-MSCM dispersed in air-exposed water for 0, 12, 24, 48, and 72 h after laser irradiation for 10 min (808 nm, 1.0 W/cm^2^). **(I)** Diameter of three BP formulations (BP NSs, BP/DACHPt, and BP/DACHPt-MSCM) dispersed in air-exposed water at the beginning (0 h). **(J)** Changes in Diameter of three BP formulations dispersed in air-exposed water for 12, 24, 48, and 72 h. Images showing the dispersibility of (1) BP NSs, (2) BP/DACHPt, and (3) BP/DACHPt-MSCM in DI water, PBS, and DMEM cell culture medium at **(K)** 0 and **(L)** 12 h.

To examine the effect of coordination with DACHPt and encapsulation with MSC membranes on the stability of BP NSs, the absorption of BP NSs, BP/DACHPt, and BP/DACHPt-MSCM solutions in air-exposed water were monitored at respective time points. At the beginning, the spectra of these three BP formulations exhibit broad absorption from UV to NIR regions (Pan, et al., 2020) (Figures 5A–C). With extended time, the absorbance of BP NSs in air-exposed water plummets (Figure 5A). After 24, 48, and 72 h of dispersion, the absorbance becomes only 80, 58 and 40%, respectively, of the original one (Figure 5D), suggesting that BP NSs rapidly and continuously degraded. In contrast, the absorbance of BP/DACHPt and BP/DACHPt-MSCM kept more stable (Figures 5B,C), with 62 and 78% retained even after 72 h, respectively (Figure 5D). Therefore, the stability in air-exposed water improved from BP, BP/DACHPt to BP/DACHPt-MSCM in sequence.

To study whether NIR irradiation destructed the surface morphology of BP/DACHPt-MSCM, the morphology of BP/DACHPt-MSCM after NIR irradiation was observed by TEM. As showed in Figure 5E, the membranes still completely covered the BP NSs, indicating that the BP/DACHPt-MSCM could be stable with no change in the surface morphology under NIR irradiation.

In consideration of the BP, formulations would undergo a period of blood circulation under physiological conditions before performing photothermal therapy, the temperature changes at respective time points of BP NSs, BP/DACHPt, and BP/DACHPt-MSCM solutions in air-exposed water after irradiating by 808 nm NIR laser (power density: 1.0 W/cm^2^, duration: 10 min) were recorded using an IR thermal camera. In the case of BP NSs, the temperature rose by 21.5°C after irradiation in the beginning of the dispersion, but only by 7°C at 12 h time point. After 72°h, irradiation barely raised the temperature, indicating the attenuation of photothermal performance along with BP NSs degradation (Figure 5F). In contrast, BP/DACHPt was more photothermally stable, allowing a temperature increase by 18.2°C after 12°h, but the temperature rise decreased markedly in the next three days, only 3.7°C after 72 h (Figure 5G). Interestingly, encapsulation with MSC membranes significantly improved the photothermal stability of BP/DACHPt, enabling the temperature increase by 19°C after 72 h (Figure 5H). Collectively, this dual protection of DACHPt coordination and MSC membrane coating significantly improved the photothermal stability of BP NSs.

To test the changes in the hydration particle size of BP NSs, BP/DACHPt, and BP/DACHPt-MSCM for 72°h, the hydration particle size of these three BP formulations in air-exposed water were measured at each time point. As shown in Figure 5I, the particle diameter of freshly prepared three BP formulations were about 205.6, 213.8, and 252.3°nm, respectively. With an extended time, the diameter of BP NSs increased continuously (Figure 5J). After 72 h of dispersion, the diameter increased by about 42.5°nm, suggesting that BP NSs markedly and continuously aggregated in air-exposed water. In contrast, the diameter of BP/DACHPt kept more stable, with increasing by about just 12.3 nm after 72°h, manifesting that the BP NSs was much more likely to aggregate than the BP/DACHPt in air-exposed DI water. For BP/DACHPt-MSCM, the increase of particle diameter was maintained below 7 nm within 72°h, especially after 48 and 72°h, the changes of particle diameter were less than those of BP/DACHPt, showing good stability of BP/DACHPt-MSCM in air-exposed water.

Next, we examined the dispersity of BP/DACHPt-MSCM nanoparticles after standing in DI water, PBS, and DMEM containing 10% FBS. As shown in Figures 5K,L, the three BP formulations (BP NSs, BP/DACHPt, and BP/DACHPt-MSCM) were all well dispersed in DI water within 12 h. Whereas in the salt solution, BP NSs and BP/DACHPt quickly aggregated, BP/DACHPt-MSCM remained homogeneous, which could be attributed to the stabilizing effect of glycans on the hydrophilic surface of biological membranes (Wu, et al., 2021). Moreover, we can see that in a DMEM cell culture medium containing 10% FBS, BP/DACHPt was much more likely to aggregate than BP NSs and the aggregation of BP/DACHPt-MSCM was more slowly than that of BP/DACHPt, indicating that membrane coating can inhibit BP/DACHPt aggregation in a DMEM cell culture medium containing 10% FBS. All these results indicated that the MSC membrane coating could significantly improve the dispersity of BP nanomaterials, which is profitable for its subsequent bio-effects.

### Cellular Uptake Assessments of BP/DACHPt-MSCM Nanoparticles

**FIGURE 6:**
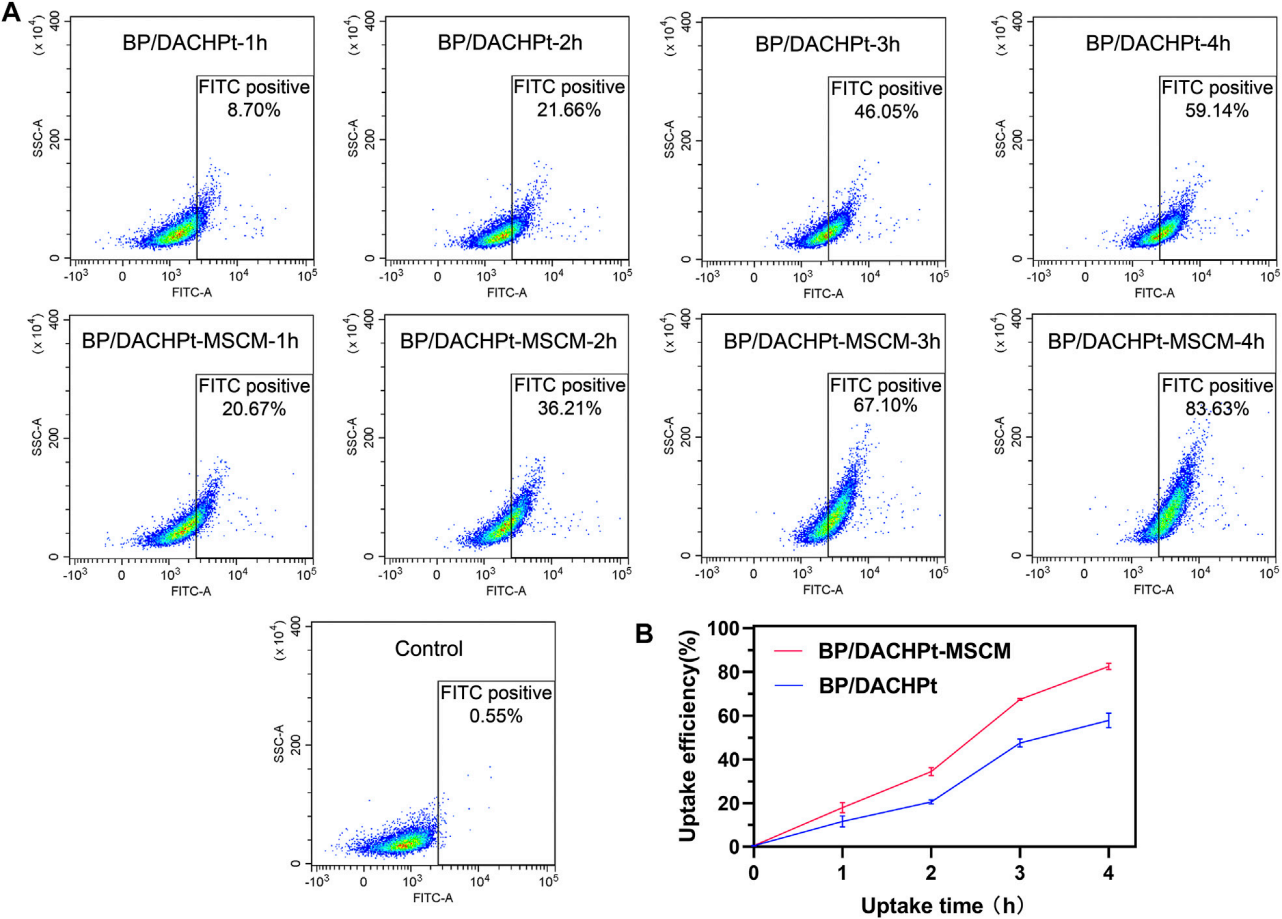
Flow cytometry (FACS) analysis of BP/DACHPt-MSCM nanoparticles for tumor cell targeting. **(A)** Pseudocolor plot of A549 cells cultured with BP/DACHPt or BP/DACHPt-MSCM for 1, 2, 3, and 4 h. The A549 cells incubated with none nanoparticles were used as control. **(B)** Time-course of the mean fluorescence intensity of A549 cell after incubation with different nanoparticles. Data are presented as mean ± s.d. (*n* = 3).

Next, we examined the camouflage effect of MSC membranes on the cellular uptake of BP/DACHPt. For this, the nanoparticles were incubated with the A549 cells, and internalization was analyzed by flow cytometry. As shown in Figure 6A, after incubation for the same time (1, 2, 3, or 4 h), the amount of internalized BP/DACHPt-MSCM was higher than that of BP/DACHPt. Figure 6B showed the time-course of the mean fluorescence intensity of A549 cell after incubation with different nanoparticles. We can see that, within 4°h, the difference between BP/DACHPt and BP/DACHPt-MSCM became more evident with an increase in incubation time. It indicated that surface coating with MSC membranes greatly increased the cellular uptake of BP/DACHPt. This suggested that MSC membrane-based camouflage significantly improved the transduction efficiency of BP/DACHPt nanoparticles for specific targeting to the cancer cells.

### Photothermal-Chemo Antitumor Efficacy of BP/DACHPt-MSCM

**FIGURE 7:**
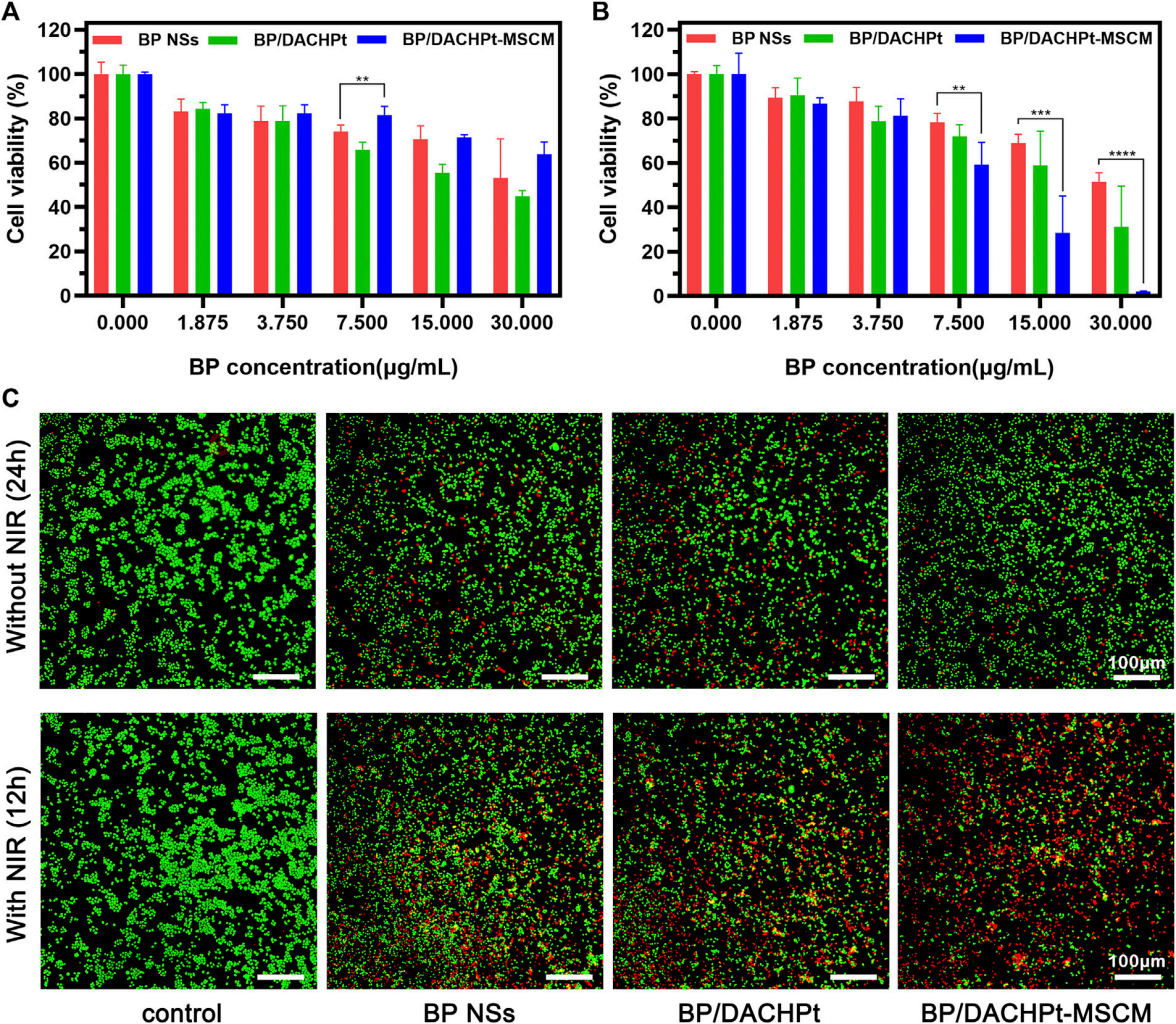
Photothermal and chemotherapeutic effects of BP/DACHPt-MSCM. Cell viability of A549 cells cultured with BP NSs, BP/DACHPt, and BP/DACHPt-MSCM **(A)** for 24 h in the absence or **(B)** for 12 h in the presence of NIR irradiation. **(C)** Fluorescence images of the Calcein-AM (green, live cells) and PI (red, dead cells) co-stained A549 cells in the absence of NIR irradiation, following incubation with BP, BP/DACHPt, or BP/DACHPt-MSCM (having internal BP concentration of 7.5 μg/ml) for 24 h or in the presence of NIR irradiation, following incubation with these three BP formulations (having internal BP concentration of 15.0 μg/ml) for 12 h (data are presented as mean ± s.d., **p < 0.01, ***p < 0.001, ****p < 0.0001).

To assess the antitumor efficacy of BP/DACHPt-MSCM, we tested the toxic effects of BP NSs, BP/DACHPt, and BP/DACHPt-MSCM (with increasing concentration) against the A549 cells. The cytotoxicity of the nanoformulations without any NIR irradiation within 24 h was tested by the CCK-8 assay (Figure 7A). We found that the nanoparticles showed cytotoxicity against the A549 cells in a concentration manner. Notably, the cytotoxicity of BP/DACHPt was stronger than that of BP NSs at the concentration of 7.500, 15.000, and 30.000 μg/ml, demonstrating that the DACHPt was successfully released and exerted the chemotherapeutic effects. Interestingly, the cytotoxicity of BP/DACHPt-MSCM nanoparticles was the least among the three materials tested. This may be due to the coating of cell membranes interfered certain cytotoxic action of BP NSs.

In addition, the cytotoxicity of these nanoformulations under laser irradiation was studied (Figure 7B). As expected, after 12 h of culture, the cell inhibition rate of BP/DACHPt-MSCM was the highest (∼98%; at 30.000 μg/ml), next was that of BP/DACHPt (∼69%; at 30.000 μg/ml), and that of BP NSs was relatively weak (∼48%; at 30.000 μg/ml). The results indicated that both coordination with DACHPt and encapsulation with MSC membranes could enhance the tumor cytotoxicity of BP formulations. To determine the drug interactions, we calculated the combination index (CI) with respect to experimental parameters (IC_50_). The CI of BP/DACHPt was 0.91, which was less than 1, confirming that the BP and DACHPt in BP/DACHPt have synergistic effects. Doubtlessly, the CI of BP/DACHPt-MSCM was less than 0.91, which was 0.46, showing that encapsulation with MSC could enhance the synergistic effect of BP/DACHPt. Notably, the IC_50_ values calculated from the CCK-8 assay were shown in Table 1. The concentration of BP/DACHPt was represented by that of BP in it and the mass ratio of DACHPt to BP in BP/DACHPt was 4:5.

**TABLE 1:** IC_50_ (μg/ml) values on A549 cells after incubation with different drugs for 12 h.

**BP**	**DACHPt**	**BP/DACHPt**	**BP/DACHPt-MSCM**
34.47	32.85	17.07	8.59

Subsequently, the cytotoxicity of all the three nanoformulations was also investigated by the Calcein-AM/PI assay (Figure 7C). The results were consistent with prior findings, that is, BP/DACHPt showed a better chemotherapeutic effect without NIR irradiation than BP NSs and its toxicity was lowered after coating with membranes. For the NIR irradiation group, the tumor cell killing effects of BP NSs, BP/DACHPt, and BP/DACHPt-MSCM enhanced in turn. Overall, these results showed the excellent tumor-targeted photothermal-chemo efficacy of BP/DACHPt-MSCM, which could be attributed to the combination of chemotherapy and photothermal therapies, as well as the improved stability and cellular uptake.

## Conclusion

In summary, we reported a biocompatible and tumor-targeting drug delivery system of MSC membranes coated BP NSs carrying the active species of oxaliplatin DACHPt. This system significantly improved the stability of BP NSs in air-exposed water by the dual protection of DACHPt coordination and MSC membrane coating. Moreover, the surface coating with MSC membranes effectively improved the dispersibility, tumor-targeting property, and photothermal-chemo tumor suppression of BP/DACHPt. We suggested that this strategy of drug-self-stabilization and functionalization with MSC membranes significantly improved the BP NSs applications in targeted photothermal-chemo cancer therapy.

## Data Availability

The raw data supporting the conclusions of this article will be made available by the authors, without undue reservation.
